# Non-coding RNAs Potentially Controlling Cell Cycle in the Model *Caulobacter crescentus*: A Bioinformatic Approach

**DOI:** 10.3389/fgene.2018.00164

**Published:** 2018-05-30

**Authors:** Wanassa Beroual, Matteo Brilli, Emanuele G. Biondi

**Affiliations:** ^1^Laboratoire de Chimie Bactérienne, Centre National de la Recherche Scientifique, Aix Marseille University, Marseille, France; ^2^Department of Biosciences, Pediatric Clinical Research Center “Romeo ed Enrica Invernizzi, ” University of Milan, Milan, Italy

**Keywords:** *Caulobacter crescentus*, Cell cycle regulation, ncRNAs, DNA replication, Two-component regulatory systems (TCS)

## Abstract

*Caulobacter crescentus* represents a remarkable model system to investigate global regulatory programs in bacteria. In particular, several decades of intensive study have revealed that its cell cycle is controlled by a cascade of master regulators, such as DnaA, GcrA, CcrM, and CtrA, that are responsible for the activation of functions required to progress through DNA replication, cell division and morphogenesis of polar structures (flagellum and stalk). In order to accomplish this task, several post-translational (phosphorylation and proteolysis) and transcriptional mechanisms are involved. Surprisingly, the role of non-coding RNAs (ncRNAs) in regulating the cell cycle has not been investigated. Here we describe a bioinformatic analysis that revealed that ncRNAs may well play a crucial role regulating cell cycle in *C. crescentus*. We used available prediction tools to understand which target genes may be regulated by ncRNAs in this bacterium. Furthermore, we predicted whether ncRNAs with a cell cycle regulated expression profile may be directly regulated by DnaA, GcrA, and CtrA, at the onset, during or end of the S-phase/swarmer cell, or if any of them has CcrM methylation sites in the promoter region. Our analysis suggests the existence of a potentially very important network of ncRNAs regulated by or regulating well-known cell cycle genes in *C. crescentus*. Our hypothesis is that ncRNAs are intimately connected to the known regulatory network, playing a crucial modulatory role in cell cycle progression.

## Introduction

In the last two decades, bacterial non-coding RNAs (ncRNAs) and small RNAs in particular (sRNAs, 50–400 nts), have emerged as central regulators of important cellular processes (Dutta and Srivastava, 2018). Most sRNAs are post-transcriptional regulators positively or negatively affecting the translation and/or stability of their targets. As a result, sRNAs have been shown to play key roles in the adaptive response to the environment and to stress conditions, in particular. In *Enterobacteria*, most of the proficient sRNAs/target pairs requires the RNA chaperone Hfq that both stabilizes them and facilitates the RNA duplex formation; however, this role of Hfq in sRNA control of gene expression is not conserved in all bacteria that possess an Hfq homolog (Vogel and Luisi, 2011). While sRNAs are best characterized in enterobacteria (*Escherichia coli* and *Salmonella*) and a few other well studied species (e.g., *Staphylococcus, Sinorhizobium, Bacillus*, and *Listeria*), transcriptomic analyses performed in extremely diverse species indicate that ncRNAs exist in virtually all bacteria, and their characterization in those other species is still a challenge today (Barquist and Vogel, 2015). Clearly the ability to know and master the activity of ncRNAs targeting specific function(s) can pave the way to the development of new precisely targeted weapons against pathogens, a pressing issue given that most of them are increasingly resistant to most known antibiotics.

**Figure 1:**
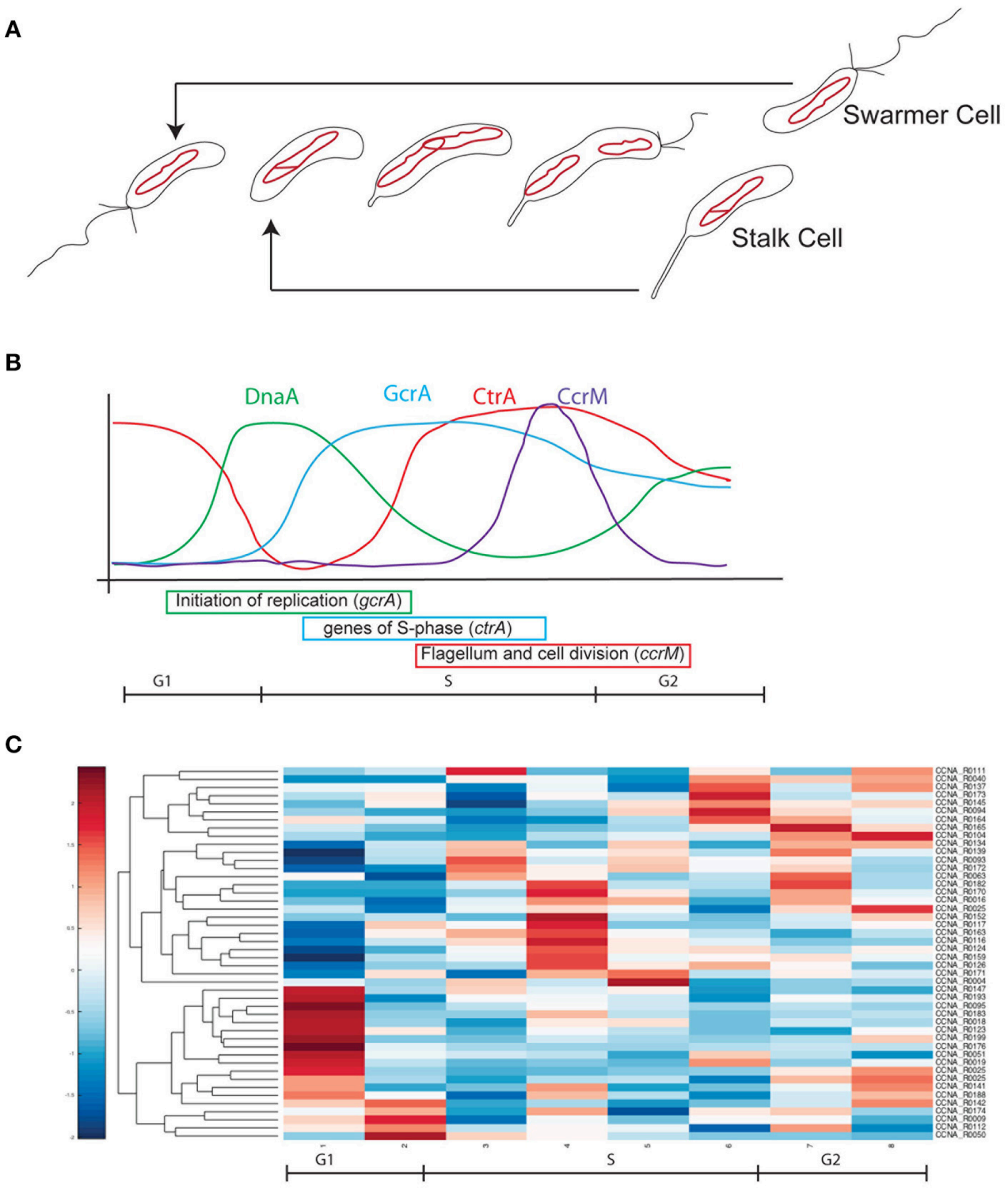
Cell cycle progression in ***Caulobacter crescentus***. **(A)** At every cell division cells divide in two different cell types, a swarmer cell (G1) and a stalked cell. Swarmer cell is unable to replicate the DNA but it is the only form able to move, as it possesses a flagellum and pili. When the swarmer cell finds a suitable environment it differentiates in a stalked cell, loosing the flagellum, retracting the pili and synthetizing a stalk at the former flagellated pole. After differentiation cells are able to initiate a single round of replication of DNA (S-phase) until a new swarmer pole is formed and cells divide at the end of a short G2 phase. The single circular chromosome of *C. crescentus* is indicated as a red circle. **(B)** This remarkable cell cycle progression is under the control of several master regulators responsible for the cell cycle regulated expression of hundreds of genes (DnaA, CtrA, CcrM, and GcrA). Protein levels are here represented in different colors. **(C)** Based on Table 1 data, ncRNAs were organized by their expression levels (Schrader et al., 2014; Zhou et al., 2015).

*Caulobacter crescentus* is a model system to investigate global regulation mechanisms such as the bacterial cell cycle and differentiation/morphogenesis (Lasker et al., 2016). This alphaproteobacterium produces a swarmer non-replicative motile cell and a sessile replicative stalked cell at every round of cell division (Figure 1A) explaining why it became the model organism in a number of top level laboratories around the world. The remarkable regulatory program implementing the cell cycle/differentiation can be easily investigated in *C. crescentus*, as a large number of pure swarmer cells in the G1 phase can be isolated and studied while in synchrony (Schrader and Shapiro, 2015). This differentiation program is under the control of a set of regulators that implement a finely orchestrated genetic circuit (Figure 1B). In *C. crescentus*, swarmer cells differentiate in stalk cells and fire a single round of DNA replication thanks to the protein DnaA, which binds the unique origin of replication and activates the DNA polymerase complex (Collier, 2012; Felletti et al., 2018). Besides controlling the replication of the chromosome, DnaA controls the transcription of the gene encoding GcrA, which in turn controls many essential genes during S-phase, including *ctrA* (Holtzendorff et al., 2004; Hottes et al., 2005; Collier et al., 2006). This latter gene codes for a response regulator that activates cell division and the expression of genes essential for cell differentiation, such as those for flagellum/pilum assembly, chemotaxis, stalk biogenesis and many others (Reisenauer et al., 1999; Jones et al., 2001; Laub et al., 2002; Biondi et al., 2006). DnaA, GcrA and CtrA constitute an essential transcriptional cascade that requires multiple regulatory levels in order to ensure the correct timing of ensuing events. For example, GcrA activity depends on methylation of the chromosome by the methyl-transferase CcrM (Fioravanti et al., 2013; Murray et al., 2013; Mohapatra et al., 2014; Haakonsen et al., 2015); CtrA's activity is regulated by phosphorylation (Biondi et al., 2006) and inactivated by a ClpXP-dependent degradation (Ryan et al., 2002, 2004; Joshi et al., 2015). Among well-known regulatory layers, surprisingly, the activity and the role of ncRNAs in *C. crescentus* has been poorly investigated, and still, they represent ideal candidates for such regulations, as they provide dynamic patterns not easily achievable with transcriptional regulation only. In 2008, a paper entitled “Small non-coding RNAs in *Caulobacter crescentus*” described 27 ncRNAs in this organism (Landt et al., 2008). Unfortunately only few of them appeared to be involved in important functions and, besides a work in 2010 describing CrfA (Landt et al., 2010), an sRNA involved in adaptation to carbon starvation, no other ncRNA identified in 2008 was further characterized. More recently another sRNA was characterized in *C. crescentus* and named GsrN (Tien et al., 2017). This ncRNA is involved in multiple stresses response factor directly controlled by the general stress sigma factor, σ^T^. Finally new recent approaches using RNAseq and post-genomic techniques expanded the *plethora* of ncRNA candidates in this species to over 100 (Zhou et al., 2015).

This observation prompted us to start a systematic investigation of this new ncRNA world aiming to identify global regulators and ncRNAs possibly involved in the cell cycle in *C. crescentus*. We applied several bioinformatics tools in order to understand what kind of functions may be controlled by ncRNAs and we focused on uncharacterized ncRNAs with a changing expression level during cell cycle. We predicted their targets in the *C. crescentus* genome and we integrate this information with motif scanning and ChIP-Seq data.

## Methods

### Prediction of targets of ncRNAs

Sequences of ncRNAs were retrieved by the annotation of genes by previous results (Schrader et al., 2014; Zhou et al., 2015). PredatorRNA (Eggenhofer et al., 2011). was used using the genome NC_011916, as deposited in the PredatorRNA website, and prediction was performed as default. TargetRNA2 (Kery et al., 2014) was performed using the following settings: NTs before start codon 80, NTs after start codon 20, Seed length 7, sRNA conservation and accessibility true, sRNA window size 13, mRNA structural accessibility true, Interaction region 20, Filter size 1,000, *P*-value threshold 0.5 (predictions were considered significant only with a *P* < 0.05). Finally CopraRNA (Wright et al., 2014) was used with default settings.

Secondary structure prediction of ncRNAs was performed using mFold (Zuker, 2003) and RNAfold (Gruber et al., 2008) using default settings.

### Prediction of DnaA, CcrM, and CtrA consensus sequences

Prections of DnaA, CcrM, and CtrA potential controls were performed as previously described (Brilli et al., 2010). Position Weight Matrices (PWM) modeling the CtrA and DnaA binding sites were used to scan the entire genome by calculating a measure of similarity for each genome position with the formula:

$$\begin{array}{l} S_{i:i+L}=\;\frac{1}{L}\overset{i+L}{\underset{n=i}{\sum }}\left(2+\log_{2}f_{nx}\right). \end{array}$$

From Schneider et al. (1986). Basically, for each position n in a genomic window of length L, where L is also the length of the transcription factor binding motif, we sum the logarithm base 2 of the frequency of nucleotide x at position n of the motif and then we average over all nucleotides.

As this score is continuous, there is the need to establish a threshold, that can be quite arbitrary. In this context, we normalize all the scores with respect to the maximum score attainable by the PWM under analysis, and we only retain scores that are at least 60% of the maximum.

ChIPseq data of GcrA were analyzed as previously described (Fioravanti et al., 2013) using the new annotation of ncRNAs (Zhou et al., 2015).

## Results

### Selection of cell cycle regulated ncRNAs

Previous works based on total RNA sequencing and 5′-RACE have identified a list of predicted ncRNAs expressed in culture conditions (Schrader et al., 2014; Zhou et al., 2015). Based on this experimental analysis we recovered all sequences previously identified and we initially separated ncRNA candidates based on previous annotation and their dynamic regulation during cell cycle. Out of 199, identified as ncRNAs expressed in rich or poor media, respectively PYE and M2G, 88 were further characterized for their Transcriptional Start Site (TSS), with few of them having multiple transcription start sites (Zhou et al., 2015). Among those ncRNAs, 43 were related to the translational machinery (tRNAs or ribosome-related), therefore we excluded them in the following analyses, as well as the TmRNA (Keiler and Shapiro, 2003; Cheng and Keiler, 2009; Russell and Keiler, 2009). The bibliographical data mining therefore allowed us to select 44 ncRNAs for further analysis. Among them, as previously described in the introduction, two ncRNAs were already characterized and named CrfA (Landt et al., 2008, 2010) and GsrN (Tien et al., 2017). In conclusion 42 ncRNAs were the object of this study (Figure 1 and Table 1).

**Table 1:** ncRNAs in *Caulobacter crescentus*.

**CCNA**	**Multiple TSS**	**Cell cycle phase G1, S, G2**	**TSS location**	**strand**	**0 min**	**20 min**	**40 min**	**60 min**	**80 min**	**100 min**	**120 min**	**140 min**	**Annotation based on Zhou et al. (2015)**
CCNA_R0004	/	/	210993	−	5.5	3.9	8.0	4.8	12.8	2.2	3.7	4.2	Cell cycle regulated
CCNA_R0009	/	/	708218	+	105.7	136.7	42.0	88.7	64.2	92.4	93.0	58.9	Small non-coding RNA
CCNA_R0016	/	/	844332	+	32.2	19.0	43.0	63.0	59.6	28.2	61.4	46.6	Small non-coding RNA
CCNA_R0019	/	/	920752	−	11.3	2.9	1.0	1.3	1.0	8.4	1.8	4.2	Stationary phase
CCNA_R0025	3	/	1176006	+	214.0	13.2	262.0	360.4	275.7	10.1	356.7	551.2	Small non-coding RNA
CCNA_R0040	/	/	1645202	+	7.8	7.9	16.0	14.3	7.7	20.0	17.9	19.4	Small non-coding RNA
CCNA_R0063	/	/	2889177	+	261.6	112.8	312.0	266.9	192.6	215.9	341.6	207.5	Minimal medium
CCNA_R0111	/	/	622892	+	0.8	1.4	5.0	0.4	0.0	2.7	0.5	3.9	Small non-coding RNA
CCNA_R0112	/	/	626656	+	121.1	143.6	101.0	118.7	109.4	66.8	139.2	74.8	Small non-coding RNA
CCNA_R0134	/	/	1294595	+	12.7	42.8	67.0	28.7	64.7	25.8	70.1	70.2	Small non-coding RNA
CCNA_R0137	/	/	1387892	−	43.3	45.3	21.0	43.5	20.0	68.8	39.8	63.5	Small non-coding RNA
CCNA_R0141	/	/	1607996	−	7.4	0.2	1.0	7.4	0.0	1.0	3.7	7.8	Small non-coding RNA
CCNA_R0142	/	/	1682820	+	13.3	16.8	4.0	6.5	3.1	5.3	8.2	15.9	Small non-coding RNA
CCNA_R0145	/	/	1911195	+	5.3	10.1	1.0	6.5	10.8	13.8	10.5	11.3	Small non-coding RNA
CCNA_R0147	/	/	1986625	+	129.1	31.7	77.0	50.4	66.2	8.2	27.9	14.5	Small non-coding RNA
CCNA_R0170	/	/	2778348	+	15.8	15.7	18.0	30.9	23.6	18.1	27.0	21.2	Small non-coding RNA
CCNA_R0171	/	/	2800063	+	0.4	5.8	0.0	7.0	8.2	3.4	5.5	3.9	Small non-coding RNA
CCNA_R0173	/	/	2967588	−	9.8	16.6	0.0	15.6	10.8	29.0	10.5	13.1	Small non-coding RNA
CCNA_R0174	/	/	2977449	+	31.6	40.9	20.0	41.7	12.3	35.9	38.9	25.4	Small non-coding RNA
CCNA_R0182	/	/	3288242	+	94.8	93.8	130.0	201.3	120.2	118.0	200.1	115.7	Small non-coding RNA
CCNA_R0183	/	/	3294320	+	17.9	1.0	1.0	10.0	2.6	0.0	1.8	2.5	Small non-coding RNA
CCNA_R0188	/	/	3462949	+	117.2	86.2	40.0	104.3	70.3	47.3	76.0	104.8	Small non-coding RNA
CCNA_R0193	/	/	3699499	+	214.6	72.0	134.0	130.4	118.6	75.3	132.8	121.0	Small non-coding RNA
CCNA_R0018	/	G1	920425	+	86.2	24.6	10.0	48.3	49.8	18.8	32.1	25.4	Rich medium
CCNA_R0051	/	G1	2404309	+	281.3	162.6	148.0	140.8	102.7	203.6	141.9	91.7	Small non-coding RNA
CCNA_R0095	/	G1	4950	−	99.3	6.2	18.0	36.1	22.6	13.5	19.2	12.7	Small non-coding RNA
CCNA_R0123	/	G1	921719	−	9.2	4.6	1.0	3.9	2.6	1.4	0.5	4.2	Small non-coding RNA
CCNA_R0176	/	G1	3091482	−	231.0	41.2	8.0	3.9	2.6	10.1	23.8	16.6	Small non-coding RNA
CCNA_R0199	/	G1	3987216	+	89.0	15.4	14.0	28.3	21.1	16.6	23.4	52.2	Small non-coding RNA
CCNA_R0025	1	G1-G2	1176026	+	139.7	81.3	59.0	83.9	86.8	49.9	131.0	150.3	Small non-coding RNA
CCNA_R0025	2	G1-G2	1176040	−	500.6	58.7	3.0	12.2	42.1	56.9	254.1	392.0	Small non-coding RNA
CCNA_R0164	/	G1-G2	2608458	−	34.3	26.2	10.0	12.2	22.6	46.8	40.8	26.8	Small non-coding RNA
CCNA_R0050	/	G1-S	2397735	+	105.5	700.9	385.0	306.0	167.9	99.2	103.9	64.2	Cell cycle regulated
CCNA_R0117	/	G1-S	772628	+	30.6	84.5	70.0	110.0	49.8	49.7	62.7	73.4	Small non-coding RNA
CCNA_R0104	/	G2	362212	−	4.5	0.2	0.0	4.8	7.7	6.0	17.4	22.6	Small non-coding RNA
CCNA_R0165	/	G2	2672357	−	25.2	10.8	2.0	10.9	18.5	42.2	78.8	43.8	Small non-coding RNA
CCNA_R0093	/	S	3781500	−	8141.7	16080.3	26189.0	16986.4	21895.1	19086.7	20603.8	15762.8	Minimal medium
CCNA_R0094	/	S	182	−	55.0	15.4	2.0	46.1	181.3	314.6	176.3	118.9	Small non-coding RNA
CCNA_R0116	/	S	757263	−	11.1	276.7	480.0	793.3	448.8	325.5	180.9	190.9	Small non-coding RNA
CCNA_R0124	/	S	938932	+	98.9	180.7	268.0	390.4	297.3	309.6	220.7	290.8	Small non-coding RNA
CCNA_R0126	/	S	1081336	−	1.4	5.5	6.0	15.6	10.3	12.8	9.6	4.9	Small non-coding RNA
CCNA_R0139	/	S	1523958	+	5.7	16.9	25.0	19.6	22.6	16.6	27.9	18.7	Small non-coding RNA
CCNA_R0152	/	S	2086425	+	447.7	526.2	416.0	1091.9	547.4	439.4	580.6	777.0	Small non-coding RNA
CCNA_R0159	/	S	2434068	+	1.0	12.3	15.0	25.6	14.9	16.9	18.3	15.2	Small non-coding RNA
CCNA_R0163	/	S	2560042	−	4.9	84.7	108.0	134.3	78.0	56.0	30.2	55.0	Small non-coding RNA
CCNA_R0172	/	S	2940670	−	51.7	71.5	210.0	159.5	181.3	146.0	173.5	109.0	Small non-coding RNA

*Coordinates of the Transcriptional Start Site (TSS) and classification of the cell cycle regulated phase are listed*.

By studying the expression patterns of the selected ncRNA in synchronized cells, we classified those 42 ncRNAs based on their expression levels during cell cycle. Specifically, we identified 23 ncRNAs whose expression changes during the cell cycle: 6 are expressed in G1, 2 at the onset of the S phase (noted as G1-S), CCNA_R0025 and CCNA_R0164 are expressed in G1 and G2 phases, while 10 are expressed in S-phase (noted as S) and 2 are toward the end of the cell cycle (G2). The latter genes may be reflecting the accumulation in a specific cell type, as we will discuss later. The observation that some ncRNAS genes are cell cycle regulated, may suggest a putative function associated to regulation of functions that are required at a specific phase (Laub et al., 2000).

### Prediction of target genes regulated by ncRNAs

Although experimental validation is absolutely required to identify targets of ncRNAs, predictive tools may be useful to suggest candidate target genes and therefore give a glance about the functions regulated by a set of sRNAs. This is particularly valid in case of a systematic analysis as the experimental validation/identification of targets is not easily scalable. In order to reconstitute the whole ncRNAs network connected to cell cycle, we used three different available tools, mainly RNApredator (Eggenhofer et al., 2011) but then as confirmation also CopraRNA (Backofen et al., 2014; Wright et al., 2014) and TargetRNA2 (Kery et al., 2014). Each analysis provides specific features and it is able to predict classes of targets. By using PredatorRNA we predicted the first 100 targets that were retained for the analysis (Supplementary Table 1).

Genes coding for factors playing a role in cell cycle regulation were suggested as probable targets of some of the ncRNAs (Table 2). In particular we considered genes annotated as of cell cycle/cell division regulators. In order to evaluate the predictions, we performed an additional analysis based on the following consideration: regions that are complementary to the target RNA should be located within open regions (loops) of the ncRNA molecule and not to paired regions (stems) to ensure accessibility. Therefore, we aimed to understand *in silico* how those putative small RNAs were structured and whether targets were interacting with the same loop regions. We used RNAfold (Gruber et al., 2008) and mFold (Zuker, 2003) in order to identify loop regions that would be more accessible for the interaction with target genes.

**Table 2:** Prediction based on PredatorRNA, confirmed by Copra and/or TargetRNA2 (regions targeted by ncRNAs are defined in Supplementary Table 1).

**CCNA**	**Cell cycle phase G1, S, G2**	**Functions:**	**Significant genes (genes for “flagellum” are not listed):**
CCNA_R0004	/	Pilus, flagellum, cell division	*CpaE, maf*
CCNA_R0009	/	Flagellum	
CCNA_R0016	/	Cell cycle	*hdaA*
CCNA_R0019	/	Flagellum	
CCNA_R0025	/	Flagellum, ppGpp	*spoT*
CCNA_R0040	/	Cell division	*ftsK*
CCNA_R0063	/	Cell division	*ftsH*
CCNA_R0111	/	Flagellum, pilus, cell division	*cpaE, cpaF, ftsK*
CCNA_R0112	/	/	
CCNA_R0134	/	Chromosome partitioning, cell division	*mipZ, ftsY*
CCNA_R0137	/	/	
CCNA_R0141	/	Cell division, cell cycle	*ftsH, tipN*
CCNA_R0142	/	/	
CCNA_R0145	/	Cell cycle, flagellum	*hdaA, divL*
CCNA_R0147	/	Cell division	*ftsE*
CCNA_R0170	/	Cell cycle, flagellum	*hdaA*
CCNA_R0171	/	Cell cycle, cell division, pilus	*divJ, cpdR, mraZ, cpaD*
CCNA_R0173	/	Cell cycle	*podJ*
CCNA_R0174	/	Flagellum	
CCNA_R0182	/	/	
CCNA_R0183	/	Cell division, flagellum	*ftsI*
CCNA_R0188	/	Pilus, cell cycle	*cpaE, divL, hdaA*
CCNA_R0193	/	Flagellum	
CCNA_R0018	G1	Flagellum, cell division	*ftsW*
CCNA_R0051	G1	Flagellum	
CCNA_R0095	G1	Pilus, flagellum, cell cycle, cell division, Chromosome partitioning	*parA, cckA, ftsH, cpaE*
CCNA_R0123	G1	Cell cycle, flagellum	*dnaA*
CCNA_R0176	G1	Cell cycle	*dnaA, divJ*
CCNA_R0199	G1	Stalk	*shkA*
CCNA_R0025	G1-G2	Flagellum, ppGpp	*spoT*
CCNA_R0025	G1-G2	Flagellum, ppGpp	*spoT*
CCNA_R0164	G1-G2	Flagellum	
CCNA_R0050	G1-S	Cell cycle, flagellum	*divJ, pleD, tipN*
CCNA_R0117	G1-S	Flagellum	
CCNA_R0104	G2	Cell cycle	*dnaA,cenR*
CCNA_R0165	G2	Cell cycle, flagellum, ppGpp	*popZ, spoT*
CCNA_R0093	S	Cell cycle	*divL*
CCNA_R0094	S	Cell cycle	*dnaA*
CCNA_R0116	S	Stalk	*shkA*
CCNA_R0124	S	Cell cycle	*hdaA*
CCNA_R0126	S	/	
CCNA_R0139	S	Pilus, Flagellum, cell cycle	*cpaF, divL, tacA, pleD*
CCNA_R0152	S	/	
CCNA_R0159	S	Cell cycle	*dnaA*
CCNA_R0163	S	Cell cycle	*kidO*
CCNA_R0172	S	Cell cycle	*chpT*

We asked whether among cell cycle-regulated ncRNAs target genes were more likely being cell cycle regulators with respect to non-cell cycle regulated ncRNAs. Among the 22 ncRNAs that showed dynamic expression during the cell cycle, we found 13 out of 22 directly acting on cell cycle regulators (ca 60%), while only 7 out of 23 (30%) of the remaining ncRNAs, not showing cell cycle regulation, have targets with a role in the cell cycle. This observation suggests that ncRNAs may be indeed cell cycle-regulated as they act on functions that need to be activated only at specific phases of cell cycle progression. But can we identify whether ncRNAs are regulated by one of the master regulators of the cell cycle?

### Presence of DnaA and CtrA boxes or methylation sites upstream cell cycle-regulated ncRNAs

In order to understand how ncRNAs are regulated by the cell cycle we explored the presence of known binding sites in their promoter regions. Specifically promoters were scanned for the presence of DnaA, CcrM (GAnTC) or CtrA putative DNA binding sites (both “half” and “full” binding sites) (Ouimet and Marczynski, 2000; Brilli et al., 2010). We also analyzed the presence of GcrA binding sites using previously published Chromatin Immunoprecipitation-deep sequencing (ChIP-seq) data (Fioravanti et al., 2013; Murray et al., 2013). Those four regulators represent the core transcriptional machinery responsible for cell cycle progression and for the coordinated expression of all the cell cycle-dependent functions. The analysis revealed that many of those ncRNAs are potentially regulated by master regulators of cell cycle (Table 3). In particular ncRNAs expressed in S-phase appear to possess binding sites of master regulators, justifying their cell regulated expression. Although the expression pattern may be the result of combinatorial regulation, this enrichment of cell cycle regulators binding motifs is intriguing and asks for experimental validation.

**Table 3:** Prediction of DnaA and CtrA binding sites, GcrA ChIPseq peaks and CcrM methylation sites.

			region^*^ = TSS − 100; TSS + 50				
**Name**	**TSS position**		**CtrA Full**	**CtrA half**	**CcrM**	**DnaA**	**GcrA peaks (ChIpseq) (Fioravanti et al., 2013)**
CCNA_R0004	210993	−	0	1	0	0	0
CCNA_R0009	708218	+	0	4	0	0	1
CCNA_R0016	844332	+	0	0	0	1	0
CCNA_R0018	920425	+	0	0	0	0	0
CCNA_R0019	920752	−	0	0	0	0	1
CCNA_R0025	1176006	+	1	2	0	0	0
CCNA_R0025	1176026	+	1	2	0	0	0
CCNA_R0025	1176040	−	0	1	1	0	0
CCNA_R0040	1645202	+	0	0	2	0	0
CCNA_R0050	2397735	+	0	0	0	0	0
CCNA_R0051	2404309	+	0	1	0	0	0
CCNA_R0063	2889177	+	0	0	0	0	0
CCNA_R0093	3781500	−	0	0	0	0	1
CCNA_R0094	182	−	2	4	1	0	0
CCNA_R0095	4950	−	0	0	0	0	0
CCNA_R0104	362212	−	0	2	0	0	0
CCNA_R0111	622892	+	0	0	0	0	0
CCNA_R0112	626656	+	0	1	1	0	0
CCNA_R0116	757263	−	0	1	3	0	1
CCNA_R0117	772628	+	0	0	1	0	0
CCNA_R0123	921719	−	0	0	0	0	0
CCNA_R0124	938932	+	0	3	0	0	0
CCNA_R0126	1081336	−	0	0	1	0	0
CCNA_R0134	1294595	+	0	0	0	0	0
CCNA_R0137	1387892	−	0	1	0	0	0
CCNA_R0139	1523958	+	0	0	0	1	0
CCNA_R0141	1607996	−	0	0	0	0	0
CCNA_R0142	1682820	+	0	0	0	0	0
CCNA_R0145	1911195	+	0	0	1	0	0
CCNA_R0147	1986625	+	0	0	0	0	0
CCNA_R0152	2086425	+	0	1	2	0	0
CCNA_R0159	2434068	+	0	0	2	1	0
CCNA_R0163	2560042	−	0	1	2	0	1
CCNA_R0164	2608458	−	0	0	0	0	0
CCNA_R0165	2672357	−	0	1	2	0	0
CCNA_R0170	2778348	+	0	0	2	0	0
CCNA_R0171	2800063	+	2	4	0	0	0
CCNA_R0172	2940670	−	0	0	1	1	0
CCNA_R0173	2967588	−	0	1	0	1	0
CCNA_R0174	2977449	+	0	0	0	0	1
CCNA_R0176	3091482	−	0	0	1	0	0
CCNA_R0182	3288242	+	0	0	0	0	0
CCNA_R0183	3294320	+	0	0	0	0	0
CCNA_R0188	3462949	+	0	0	0	0	0
CCNA_R0193	3699499	+	0	0	0	1	0
CCNA_R0199	3987216	+	0	2	1	2	0

* *Opposite for genes annotated on the minus strand*.

**Figure 2:**
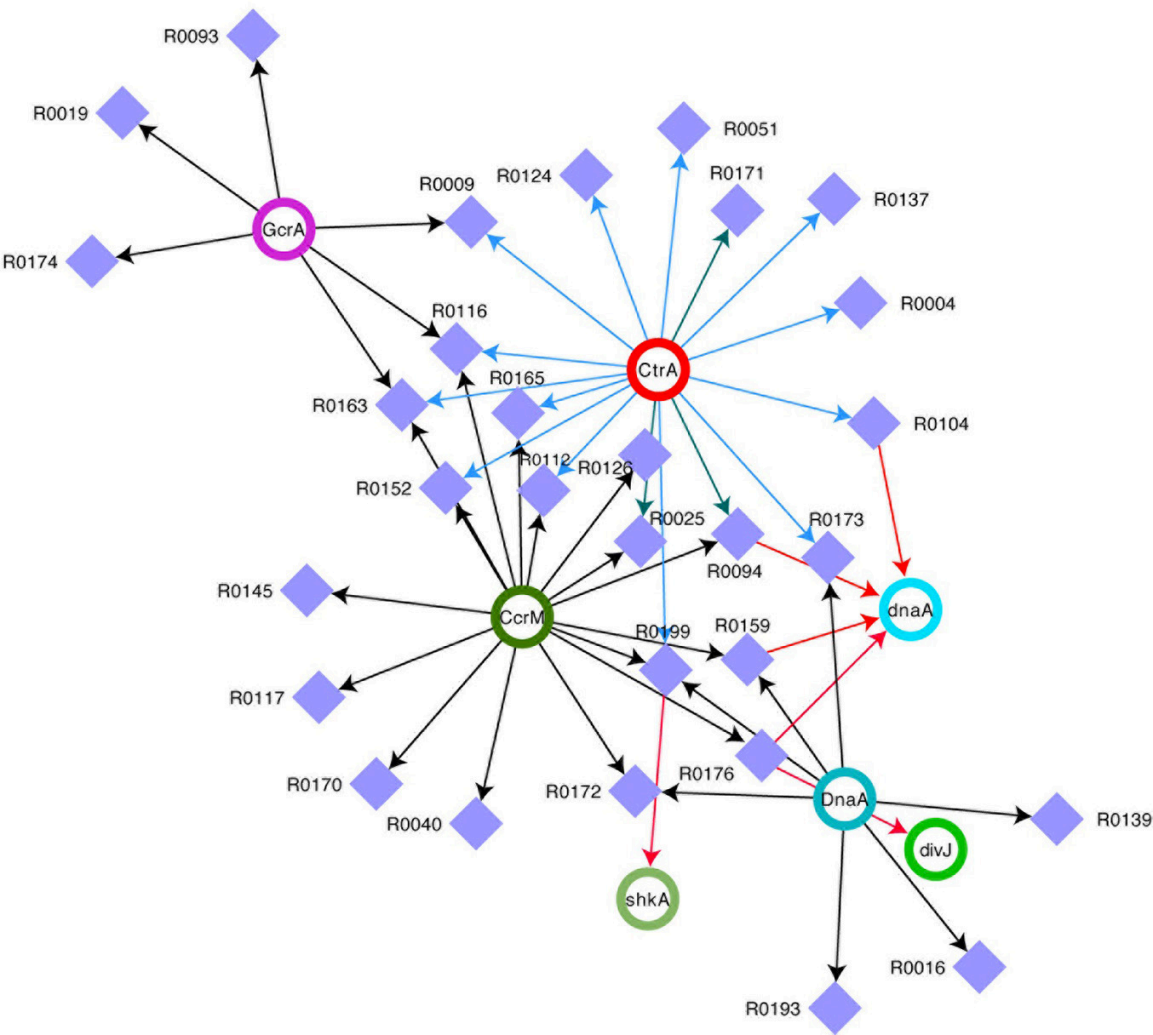
Network of ncRNAs and master regulators of cell cycle. Integrated view of control on selected ncRNAs by DnaA, CtrA, CcrM, and GcrA and of ncRNA on transcripts. Violet squares represent ncRNAs. CtrA, DnaA, CcrM, and GcrA (Upper Case) represent proteins, while the same factors (lower case) represent targets of ncRNAs. For CtrA blue arrows represent “half sites” while green arrows represent “full sites” (whenever a full site is present, we don't indicate half sites).

In order to understand the full picture of interconnection between ncRNAs and master regulators, we integrated the data of Table 2 (cell cycle targets of ncRNAs) with those in Table 3 (master regulators regulating ncRNAs) and we represent the regulations as a network (Figure 2). This network represents ncRNAs that are potentially regulated by master regulator of cell cycle and that are eventually connected to cell cycle regulator genes.

## Conclusions and perspectives

Our comprehensive analysis of *C. crescentus* ncRNAs has revealed that many of those factors potentially play important roles during the cell cycle. In particular, our predictions show that many of them target the UTRs of *dnaA* or *hdaA*. Initiation of DNA replication is a fundamental event during the cell cycle and for this reason, it likely justifies multiple regulation levels. DnaA has been shown to be subject to multiple regulatory layers (Felletti et al., 2018), including a ncRNA named SsrA, or tmRNA, a ncRNA interacting with ribosomes to regulate protein degradation (Keiler and Shapiro, 2003). Here we indicate for the first time several ncRNAs able to potentially regulate *dnaA*.

However the extent of ncRNAs regulation on the cell cycle is even vaster (Figure 2). Clearly, CtrA and CcrM seem to play a major role in coordinating the expression of many genes, while *dnaA* seems to be a major target of at least four ncRNAs. Several ncRNAs seem to be controlled by two master regulators and R0116 is controlled by CcrM, GcrA, and CtrA, suggesting an important role for this ncRNA.

Although this analysis is based on predictions and every connection must be validated experimentally, the amplitude of all connections revealed by this bioinformatic predictions strongly suggests that ncRNAs are indeed playing a major role in cell cycle regulation of *C. crescentus*. Next years will reveal whether this preliminary analysis grasped this important role.

## Author contributions

All authors listed have made a substantial, direct and intellectual contribution to the work, and approved it for publication.

### Conflict of interest statement

The authors declare that the research was conducted in the absence of any commercial or financial relationships that could be construed as a potential conflict of interest.

## Supplementary material

Supplementary Table 1 Results of predictions by RNA predator (see Materials and Methods).
